# The Role of C1-Esterase Inhibitors in the Management of Vasogenic Edema in Glioblastoma

**DOI:** 10.1155/2020/7981609

**Published:** 2020-01-28

**Authors:** Gillian R. Naro, Nicholas Noverati, Timothy Craig

**Affiliations:** Penn State University College of Medicine, Hershey, PA, USA

## Abstract

Glioblastoma (GB) is one of the most common adult primary brain tumors, classified as a grade IV astrocytoma and highly malignant in nature. As the tumor grows and disrupts the blood-brain barrier (BBB), vasogenic edema can result. The edema has the potential to significantly contribute to a patient's morbidity and mortality. Bradykinin has been theorized to play a role in this process as well as encourage tumor spread. Here we discuss a case in which a patient with vasogenic edema and angioedema refractory to antihistamines and high dose corticosteroids responded to C1-esterase inhibitor (C1INH) therapy. Though data exist concerning the role of bradykinin in GB, no clinical studies using C1INH have been done in humans with GB.

## 1. Introduction

Gliomas are the most common type of adult primary brain tumors, of which there are three subtypes: astrocytomas, oligodendrogliomas, and ependymomas. Glioblastoma resides within the category of astrocytoma and is classified as grade IV/IV according to the World Health Organization [1]. Prognosis is deemed as poor, with 5-year survival after radiation and temozolomide chemotherapy at 9.8% [2]. It is thought that mesenchymal stem cells influence glioblastoma invasiveness through cross-talk pathways that lead to an upregulation of bradykinin, a vasoactive peptide, and its receptors: bradykinin 1 receptor (B1R) and bradykinin 2 receptor (B2R) [3, 4]. B1R receptors are more highly expressed in pathological processes involving tissue damage [5], while B2R are constitutive throughout the body and when expressed on brain tissue, play a role in the regulation of the BBB [5, 6]. Gliomas have been shown to express both B1R and B2R, augmenting the tumors' vascular dilation and blood flow properties [5, 6]. Research suggests that bradykinin, particularly when acting on B2R, is also involved in the formation of vasogenic edema by disrupting the blood-brain barrier [7, 8]. Edema adds to the tumor's mass effect as it invades surrounding parenchyma, ultimately increasing intracranial pressure and contributing to overall morbidity and mortality. 

**Figure 1:**
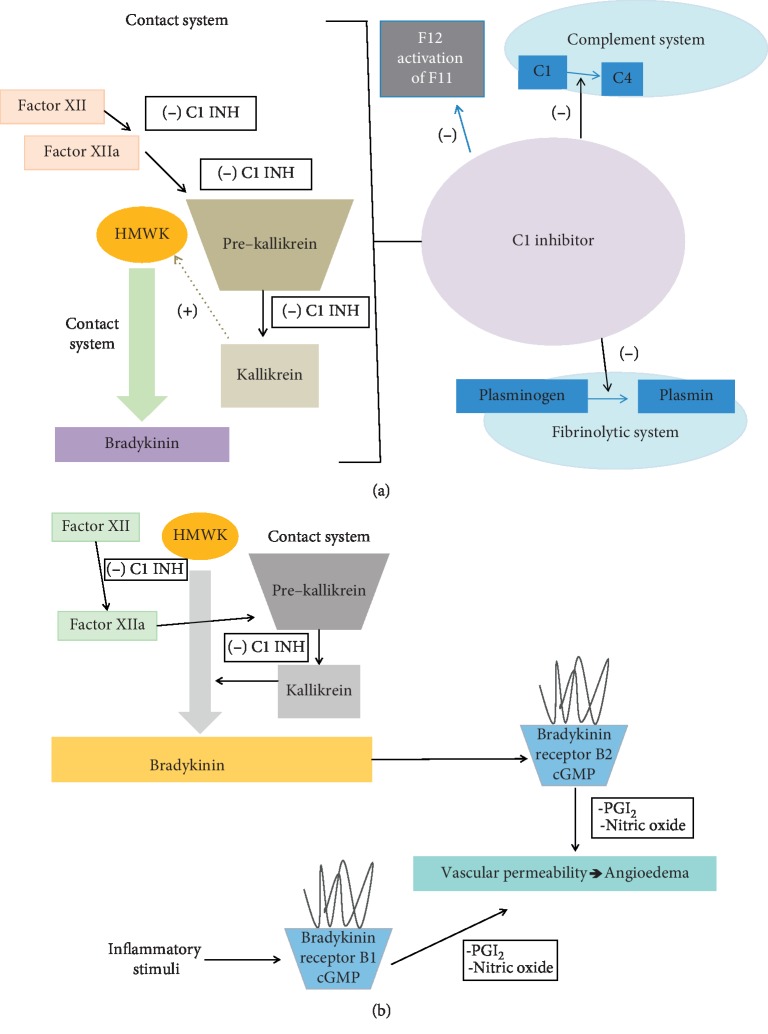
Demonstrating the contact system and how bradykinin is generated. (a) C1INH effects on coagulation, complement, contact, and fibrinolytic pathways. (b) Effect of C1INH on bradykinin and bradykinin effect on the B-1 and B-2 bradykinin receptors.

Bradykinin is generated via the contact system (Figures 1(a) and 1(b)) in response to inflammation, infection, and cancer. Bradykinin is made from the precursor high molecular weight kininogen (HMWK) via the kallikrein-kinin system [6]. When generated, bradykinin binds to the B2R. This process produces cGMP, nitric oxide, and prostacyclin that causes increased vascular permeability and associated angioedema. This process is refractory to corticosteroids and antihistamines and instead is inhibited by C1INH, kallikrien inhibitors, and bradykinin receptor antagonists. C1INH blocks the activation of factor-12, kallikrien, the complement system, the fibrinolytic system, and to a lesser extent the coagulation pathway [9].

In hereditary angioedema, C1INH is deficient, which results in recurrent angioedema of the mucosa and skin. Replacement of the C1-inhibitor reduces the recurrent symptoms, and the effect can also be measured by a return of C4 and D-dimer levels to normal [10].

Treatment of cerebral edema secondary to neoplasms usually entails treatment with corticosteroid therapy. However, for patients who have increasing edema unresponsive to steroids, there are few additional medical therapies available. This, combined with recent research that supports bradykinin's role in both tumor invasion and vasogenic edema, has led to interest in the usefulness of C1INH as a potential therapy to decrease the amount of bradykinin generated by malignant glioblastoma cells [3, 4, 11]. Our case discusses a patient with advanced glioblastoma who had diffuse angioedema refractory to steroid therapy who responded well to plasma-derived C1INH replacement. This success may help inspire future trials to explore the utility of this drug as an addition to the arsenal of available treatment modalities for highly aggressive glioblastomas.

## 2. Case Report

A 49-year-old male was evaluated on 9/26/2016 at a community emergency department for an acute headache and word-finding difficulties. Brain imaging was performed, revealing a frontal lobe tumor, and a diagnosis of glioblastoma was suspected. He underwent initial resection of his tumor on 9/30/2016 and re-resection on 11/9/2016, both of which yielded pathology confirming grade IV glioblastoma multiforme (GBM). He then underwent adjuvant temozolomide chemotherapy as well as radiation. He discontinued temozolomide on 10/6/2017 after starting erlotinib 150 mg daily and thalidomide 100 mg in July 2017. He also experienced his first seizure in March 2017 and was placed on levetiracetam for prophylaxis. Unfortunately, a new lesion was seen on repeat imaging in November 2017 which prompted initiation of lomustine on 11/28/2017; however, he only received one dose and continued on erlotinib and thalidomide.

On 1/31/2018, he developed angioedema of his lips and eyelids that lasted 3 days. This presentation was in the setting of a remote history of swelling of his larynx and multiple body sites in 2004 that was not responsive to steroids or antihistamines. For this reason, an assessment was made in an allergy and immunology clinic in March 2018, and the patient was tested for hereditary angioedema by checking C1q, C4, and C1-inhibitor protein and function. These labs were within normal limits. The patient at this time also had significantly increased vasogenic cerebral edema seen on imaging on 2/27/2018. Despite these normal labs, it was decided to try plasma-derived C1INH due to literature suggesting potential benefits to cutaneous angioedema, cerebral vasogenic edema, and possible slowing of GBM progression [3, 4, 11]. The patient was thus started on 20 units/kg infusions twice weekly on 3/13/2018. On 3/2/2018, he was seen by his hematology/oncology physician who assessed his functional status as an Eastern Cooperative Oncology Group (ECOG) score of 3.

**Figure 2:**
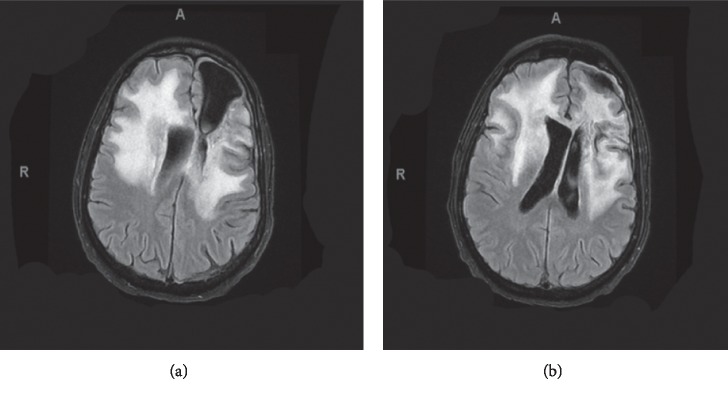
Pretherapy T2 FLAIR, March 2018. (a) and (b) demonstrating significant cerebral edema and midline shift.

**Figure 3:**
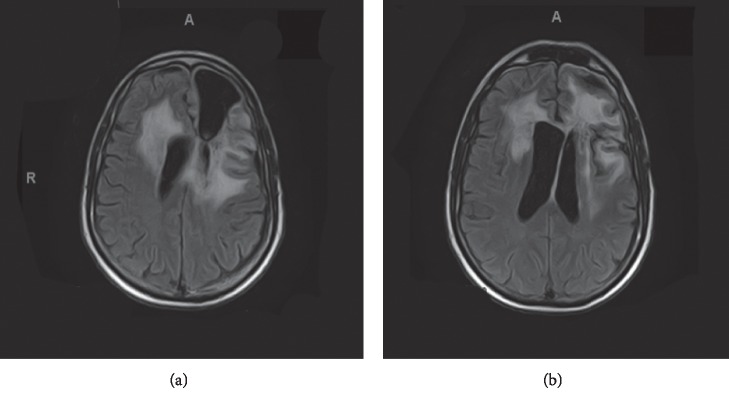
Posttherapy T2 FLAIR, May 2018. (a) and (b) showing improvements in cerebral edema seen in comparison to March 2018 imaging in Figure 2.

On 3/30/2018, the patient underwent repeat MRI brain imaging. Results showed significant decreased enhancement in frontal lesions and edema (Figures 2(a) and 2(b)). A follow-up visit also assessed his functional status as ECOG 2 on 4/5/2018. Repeat MRI brain imaging was done again on 5/18/2018 (Figures 3(a) and 3(b)), confirming continued reduced cerebral edema and midline shift; however, at this time the patient showed continued clinical decline with a worsening gait and ECOG of 3 assessed on 6/2/2018.

## 3. Discussion

Basic science literature suggests that C1 influences tumor growth in nonhuman models [12]. However, there are no controlled or small cohort human studies assessing the benefit of plasma-derived C1INH on glioblastoma progression and associated angioedema [4, 7, 13, 14]. The mechanism of how glioblastoma can activate bradykinin has been demonstrated by basic research, nonclinical studies [4, 7, 15]. Because of the extremely poor prognosis of glioblastoma, broadening the availability of effective interventions to lessen the burden of morbidity and mortality is needed. In light of the severity of our patient's CNS tumor edema, as well as his history of angioedema refractory to antihistamines and corticosteroids, combined with literature review of basic science research, we tried plasma-derived C1INH in the hopes of improving the outcome of our patient. After receiving an informed consent signed by the patient and his spouse, we treated the patient with human plasma-derived C1INH at 20 units per kg twice a week. He has tolerated the therapy well without apparent adverse effects. His cutaneous angioedema has completely resolved. It also appears his CNS edema has improved and is controlled. Although he did eventually decline clinically due to his end-stage disease, this does not discount the potential effects of this novel treatment. We are also unable to determine if the tumor was affected by the C1INH since multiple interventions were used to treat the neoplasm. However, as noted above, the literature suggests that bradykinin generation is utilized by the tumor as a spreading factor [4, 7]. Other therapies such as lanadelumab, a kallikrein inhibitor, may also be effective at controlling the edema induced by bradykinin [16, 17]. Lanadelumab is a humanized monoclonal antibody against kallikrein having an approximate 15-day half-life and thus may not only be more specific but also have less of a drug burden than C1INH. At this time, however, it seems rational to investigate C1INH since it not only inhibits bradykinin production but also can control the complement system, both of which seem to be manipulated by the tumor [3, 4, 7, 8, 18]. This case suggests that further clinical studies are needed to study bradykinin inhibition in severe refractory cases of GBM and the role that C1INH can play in the prognosis of patients with this severe disease.

## 4. Conclusion

Data suggest that glioblastoma may use elements of the innate human complement system to further its spread, produce vasogenic edema, and enhance overall tumor maintenance through inhibition of antitumor inflammatory responses [3, 4, 7, 8, 18]. Our case demonstrates how plasma-derived C1INH, through the inhibition of the contact system, appears to be a successful means of controlling cutaneous angioedema as well as CNS edema. Further research is warranted to determine if inhibition of the contact system can be a standardized therapeutic strategy for patients with glioblastoma and especially those with vasogenic edema refractory to more traditional therapies. As ongoing research explores the relationship between tumorigenesis, maintenance, and the human immune system, our case adds to the growing body of evidence to support the efficacy and safety of plasma-derived C1INH's use in GBM patients.
